# Typhoid

**Published:** 1894-06-30

**Authors:** 


### Medical Progress.

### TYPHOID—(*Continued*).

A remarkable outbreak of typhoid recently took place through the wide use of refuse or "separated" milk from a creamery which had been infected from a single farm in Ireland. It was traced to one of the milkers nursing a typhoid patient. Dr. Welply[1] points out that a streak of filth on the hands a quarter of an inch long may contain 2,500 germs, and as they double their numbers in thirty minutes, they would number about 40,000 in two hours' time if they were washed off into the milk. He adds that no less than six epidemics have occurred in twelve months caused by the infection of co-operative butter factories, and rightly considers that we have a serious danger to meet in this new system. No cases were traced by him to the use of the butter, but all to the separated milk. The difficulty of the diagnosis of typhus from typhoid and other diseases has lately been exemplified in Liverpool and the north of France.[2] Till the fourth day there is little to distinguish it from pneumonia or typhoid, but if the rash appears on the back of the hands and trunk there is greater possibility of coming to a decision. The rarity of the disease leads to its being frequently mistaken when actually met with. Another paper on the use of Ehrlich's diazo test in typhoid is contributed by W. R. Grove[3] from experiments made in Guy's Hospital. He found it thoroughly reliable if due precautions were taken. He adds 50 parts of the sulphanilic solution to one of the nitrite, and mixes with an equal quantity of urine. The product is made alkaline with solution of ammonia and shaken. In typhoid the foam is of a delicate salmon colour, but the reaction is not found in the first few days of the disease, and disappears in the third week unless a relapse occurs, when it returns. Filipovitch[4] describes as a diagnostic sign of typhoid a yellowish, orange, or saffron colour on the prominent parts of the palmar and plantar surfaces, which disappears as the patient recovers. Baczkiewitz tested the value of certain intestinal antiseptics.[5]

B-napthol with salicylate of bismuth reduced microbes in the fœces from 125,000 to 54,000 in four days, and finally to 1,800 per millegramme, and in another case from 1,860,000 to 92,000. Salol did not reduce the numbers at all, but iodole brought them down from 3,080,000 to 1,800 in the case of a diabetic patient. Tannic acid also reduced them from 470,000 to 14,000 in three days. Obraztsow of Kiev[6] gives as a distinguishing mark of typhoid the presence of a thickened tender coil of intestine in the right iliac fossa. This can be palpated by the hand held at right angles to Poupart's ligament over a spot two and four-fifth inches from the right anterior superior iliac spine. In many cases enlarged mesenteric glands may be felt near the external border of the right rectus muscle. He regards them as an unfavourable sign for the progress of the disease.

Commissioner Edson, of New York, brings facts to show that the typhoid bacillus survives in river water longer during the cold half of the year than the warm.[7] Ordinary bacteria in the water were found to decrease from 10,500 in December to 300 per centimetre in July. Hence water purifies itself most rapidly in warm weather, and typhoid outbreaks are usually in the spring or fall.

Some important investigations have been carried out on the bacteriology of typhoid. Grimbert finds that the bacillus of Eberth disappears rapidly from water if the bacillus coli is present, and promises further investigations,[8] but Uffelman has given proof of its vigorous vitality under other circumstances, for he has shown that it remains alive in garden earth three weeks, in sand nearly three months, on linen two and a half months, and on wood for a month. It is carried also in the air with dust, and is thus enabled to infect food or milk. Richmond,[10] of Owens College, in experimenting with Nicolle and Morax' stain for the flagella of bacteria, is inclined to consider that the character of the flagella afford no means of distinguishing Eberth's from the bacillus coli for they are nearly the same in each, eight to twelve in number. The difficulty in staining arises from the need of using a mordant, which precipitates other substances around the bacillus and obscures the flagella. The cholera bacilli have been already divided into three types based on the difference in their flagella. McWeeney[11] has succeeded by Parietti's method in getting pure cultivation of the typhoid bacillus from the suspected water of a village well, into which filth had been thrown. He mentioned in the discussion the view of some French authorities that the bacillus coli when grown in putrefactive media becomes the source of typhoid.

Janisewski found Eberth's bacillus in the organs of a new-born child, the offspring of a woman suffering from typhoid, but it is difficult to see what proof there is that it was infected in utero and not during or after birth.[12] Among complications lately recorded, we may note the following cases. Symptoms of colitis appeared in a patient recovering from typhoid, and on her death, three months from the beginning of the illness, extensive ulceration of the whole colon were found. The specimen was shown at the Midland Medical Society.[13] Similar severe ulceration of the colon in a patient dying in the fourth week was shown by Dr. G. P. Biggs, of New York, and another case was narrated from the Presbyterian Hospital in that city.[14]

The great number of relapses met with in the 1893 epidemic in London is discussed by H. M. Stewart. In the study of fifty typhoid cases from the records of Guy's Hospital it was found that the majority occurred after an apyretic period of five to eight days, the stages of the second attack are reached and passed more quickly than in a primary one, and that constipation is a frequent forerunner of them. He believes the cause of relapses to be the infection of healthy follicles by the passage of sloughs over them, and that therefore the large intestine is the part chiefly attacked in relapses.[15]

Typhoid bacilli have now been shown to remain alive in sterilised soil and dust for a considerable time by the recent investigations of Dr. Dempster[16]. In dry sand they were alive on the eighth day, in dry earth on the seventeenth day, in moist white sand on the twenty-third day, and in moist earth on the forty-second day. In peat they died in twenty-four hours. No fresh knowledge has been gained, however, as to their life in unsterilised soils.

[1] Lancet, April 21, 1894. [2] B.M.J., March 17, 1894. [3] Practitioner, March, 1894. [4] Am. Med. and S. Bulletin, February 1, 1894; [5] Am. Med. and S. Bulletin, February 1, 1894. [6] Med. Week, Jan. 19, 1894. [7] Med. Rec., May 5, 1894. [8] Med. Week, May 18, 1894. [9] B.M.J., May 5, 1894. [10] B.M.J., April 28, 1894. [11] Lancet, May 19, 1894. [12] Presse Med., March 24, 1894. [13] Lancet, March 10, 1894, [14] Med. Rec., April 28, 1894. [15] Practitioner, March, 1894. [16] Med. Week, May 25th, 1894.

